# Supplementary material for: Global emergence of a hypervirulent carbapenem-resistant Escherichia coli ST410 clone
Source: Nat Commun. 2024 Jan 12;15:494. doi: 10.1038/s41467-023-43854-3 (PMC10786849; doi:10.1038/s41467-023-43854-3)
Supplement: Supplementary file 1 — Supplementary Information [file 41467_2023_43854_MOESM1_ESM.pdf]

Supplementary information for:

Global emergence of a hypervirulent carbapenem-resistant *Escherichia coli* ST410 clone

Xiaoliang Ba[1#], Yingyi Guo[2#], Robert A. Moran[3], Emma L. Doughty[3], Baomo Liu[4], Likang Yao[2], Jiahui Li[2], Nanhao He[2], Siquan Shen[5,6], Yang Li[7], Willem van Schaik[3], Alan McNally[3], Mark A Holmes[1*] and Chao Zhuo[2*]

This file contains supplementary figures S1-S10, and supplementary tables S1-S4.

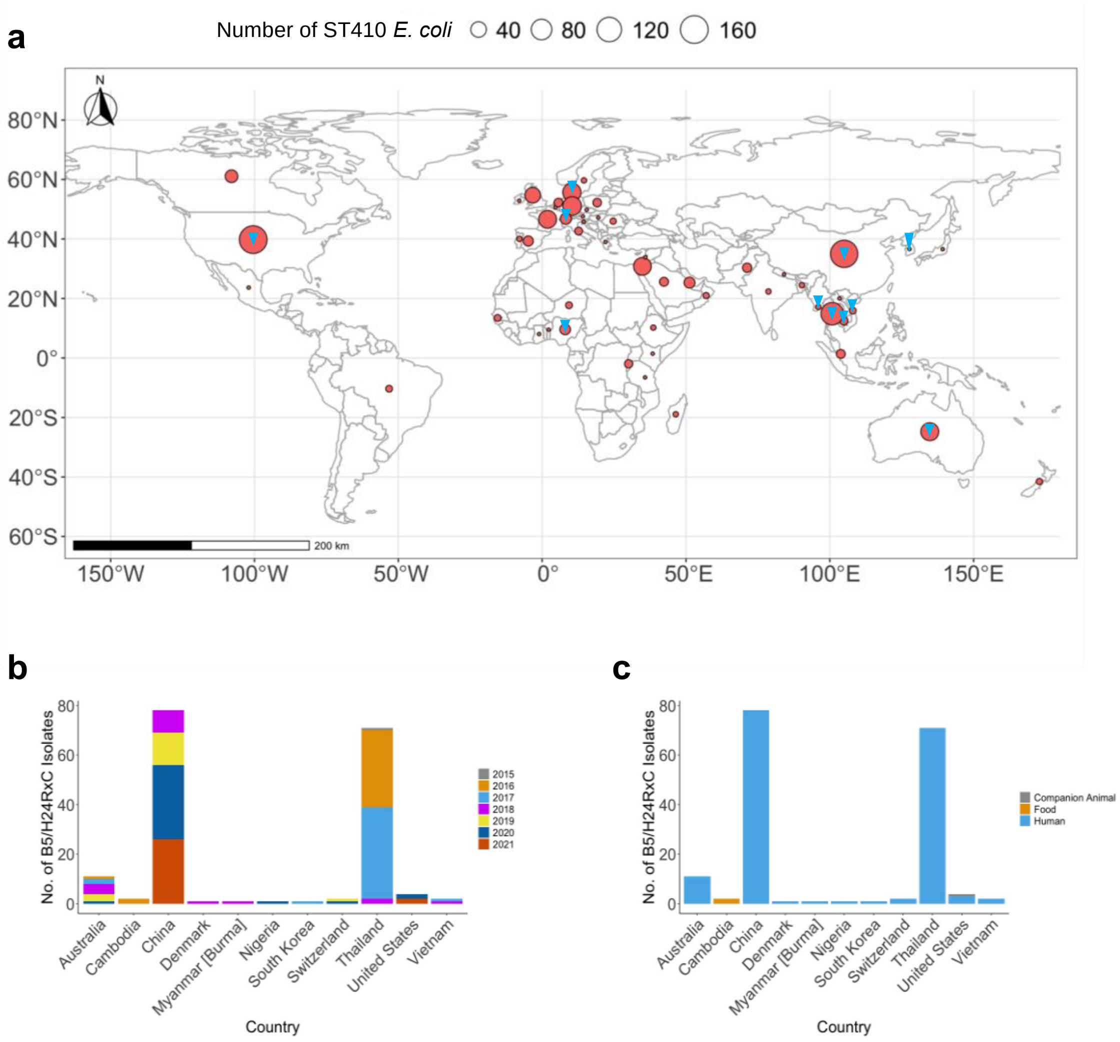

Fig. S1: Distribution of the international collection of ST410 (n=956) and B5/H24RxC clone (n=174). (a) Total number of *E. coli* in each country is represented with red circles, and blue inverted triangles indicate the presence of the B5/H24RxC clone. **(b)** Bar chart showing numbers of B5/H24RxC isolates in each country; year of isolation was coloured coded. **(c)** Bar chart showing numbers of B5/H24RxC isolates from each source. Source data are provided as a Source Data file.

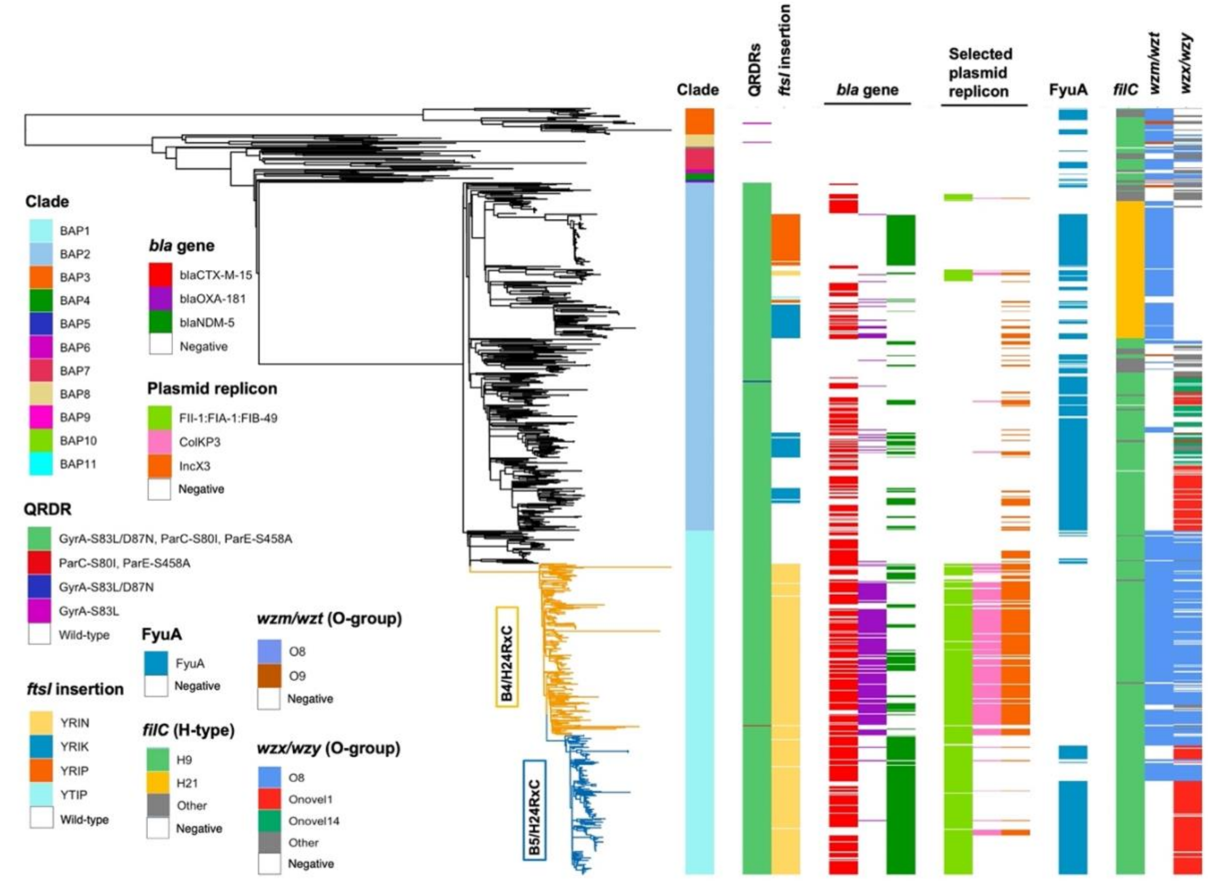

Fig. S2: Phylogeny of a global ST410 collection. Midpoint rooted maximum-likelihood phylogeny of 956 global ST410 was constructed using a core genome SNP alignment generated by Snippy v4.6.0 with ST410 isolate YD786 (GenBank accession CP013112.1) as the reference. Branch support was performed with 1,000 bootstrap replicates. QRDRs mutations and FtsI insertion that are used to define ST410-B2 and ST410-B3 are presented in the second and third columns[1]. *bla*$_{CTX-M-15}$ and *bla*$_{OXA-181}$ used to define B3/H24R and B4/H24RxC according to the Roer *et al.*[2] classification are shown in the fourth and fifth columns. *fyuA*, the indicative gene for the presence of the high pathogenicity island (HPI) is also presented. Source data are provided as a Source Data file.

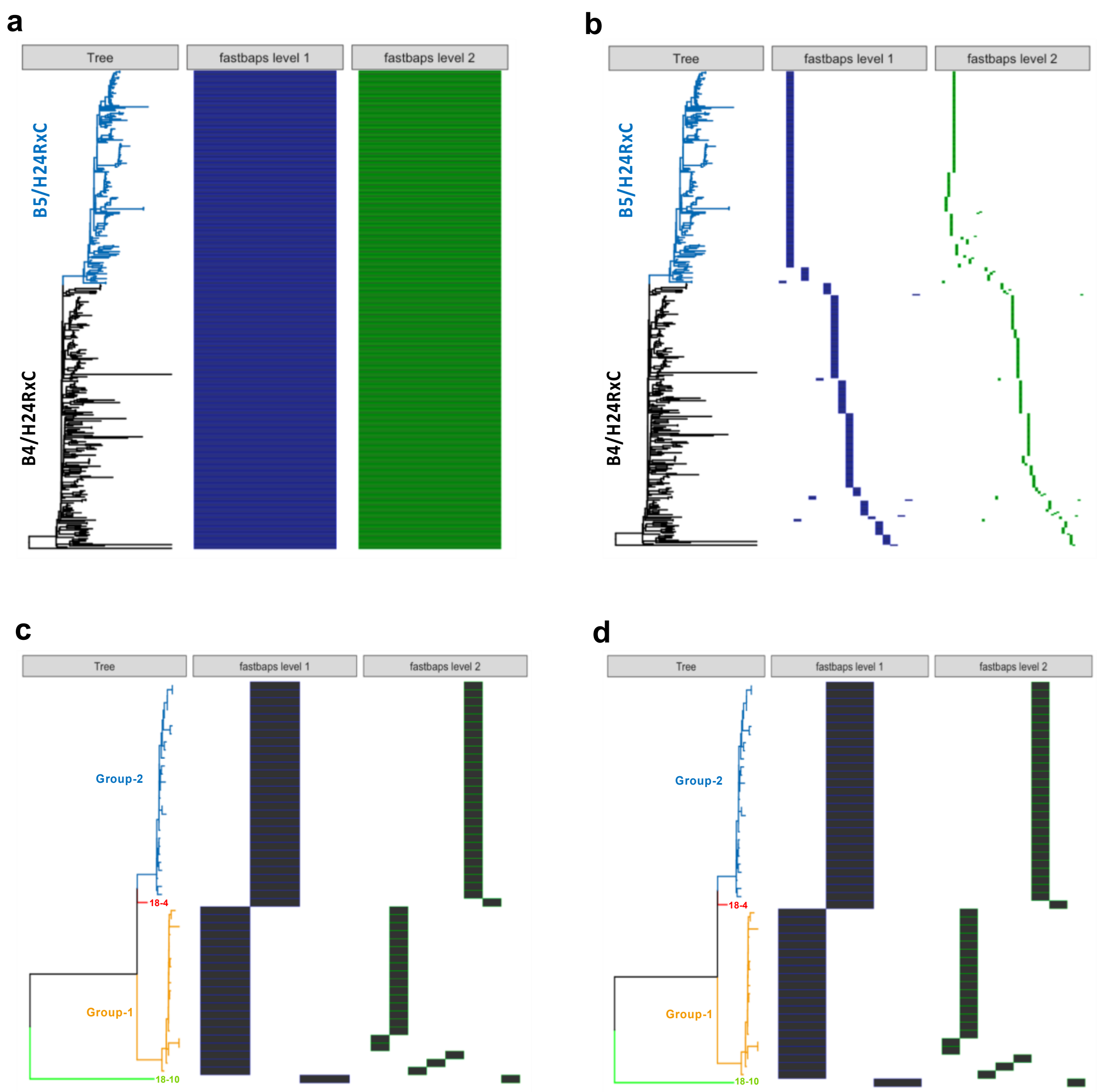

Fig. S3: Analysis of population structure within the maximum-likelihood phylogeny was conducted using Fastbaps. (a) and **(b)** used the same phylogeny (for B4/H24RxC and B5/H24RxC only) but **(a)** used Gubbins-filtered polymorphic sites generated by Snippy, while **(b)** used unfiltered core-SNPs alignment. **(c)** and **(d)** used the same phylogeny (for ST410 isolates in the children's hospital only) but **(c)** used Gubbins-filtered polymorphic sites generated by Snippy, while **(d)** used unfiltered core-SNPs alignment.

a

b

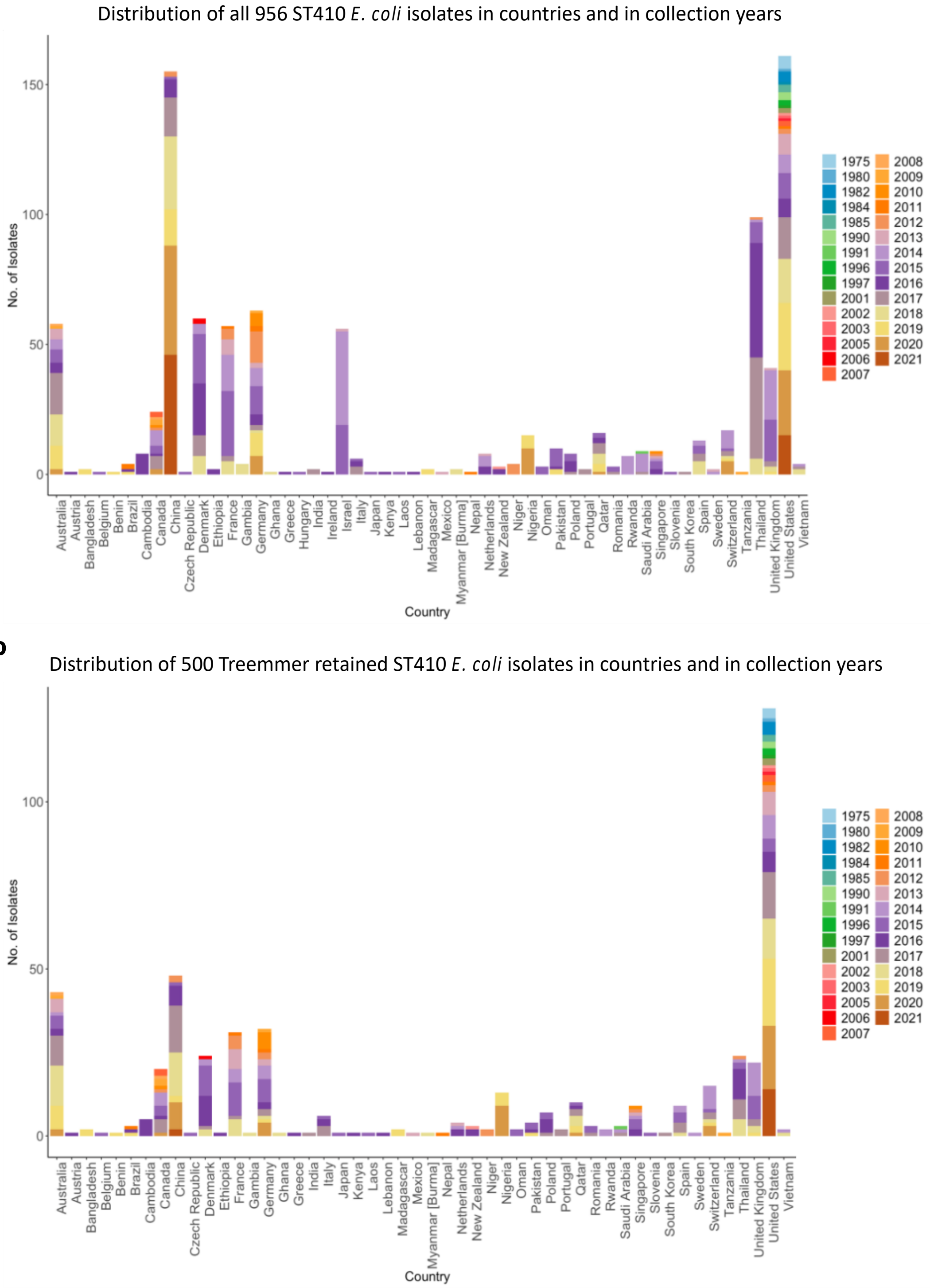

Fig. S4: (a) Bar chart showing the distribution of all 956 ST410 *E. coli* isolates in countries and in collection years. **(b)** bar chart showing the distribution of Treemmer retained ST410 *E. coli* isolates (n=500) in countries and in collection years. Source data are provided as a Source Data file.

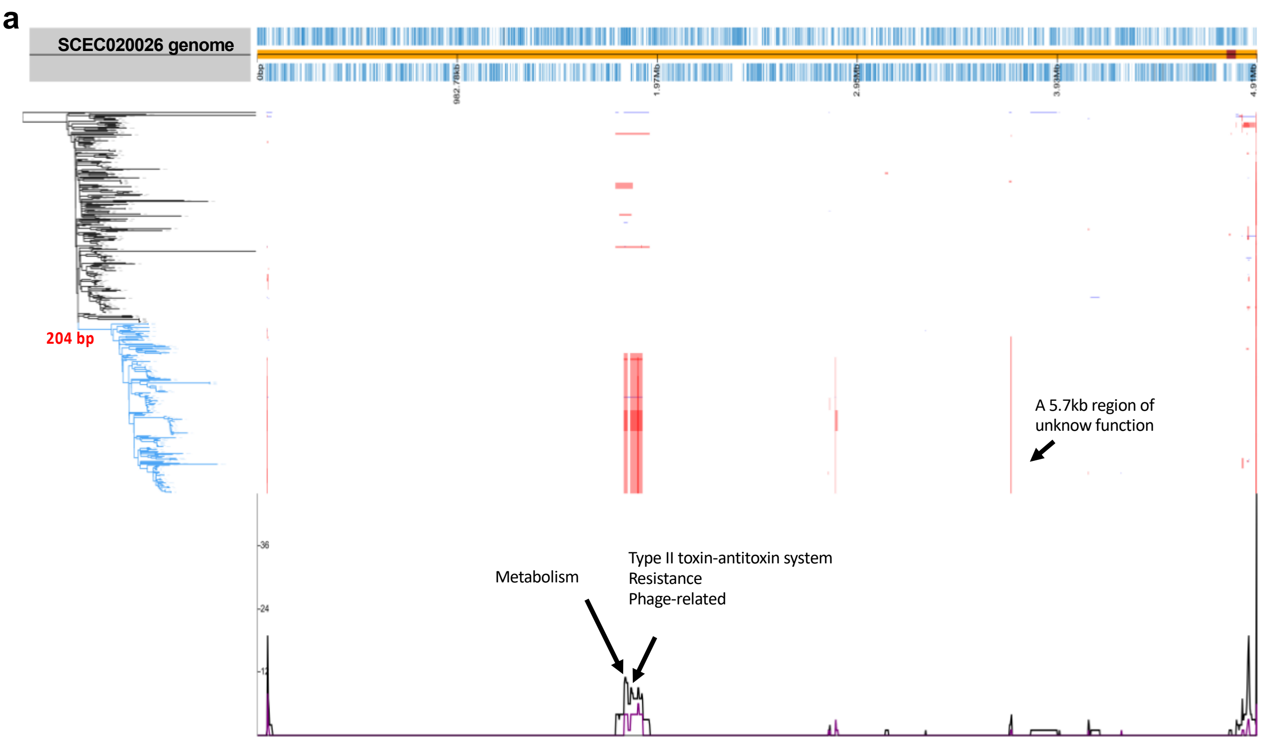

Fig. S5: Identification of recombination regions in ST410 clones B4/H24RxC and B5/H24RxC. The B4/H24RxC clone is in black and the B5/H24RxC clone is in cyan in the phylogeny. Phandango[3] was used for visualisation.

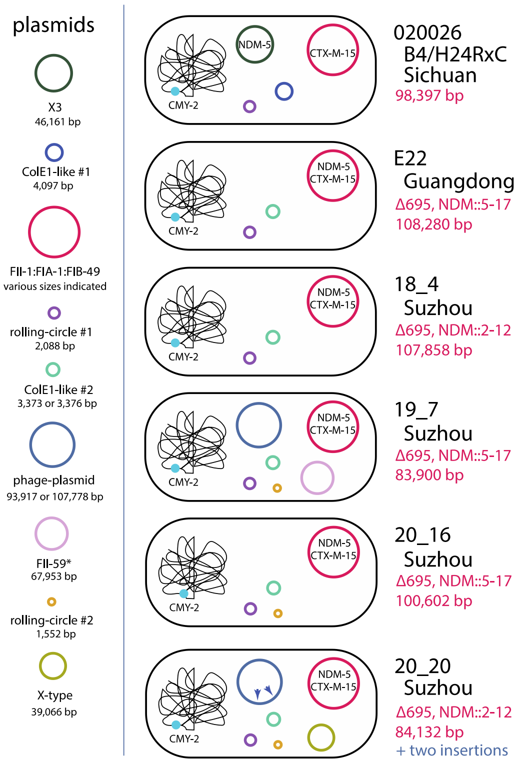

Fig. S6: Schematic presentation of the chromosome and plasmids in the B4/H24RxC isolate 020026 and different B5/H24RxC isolates. B4/H24RxC 020026 was reported previously (Genbank accessions CP034954 to CP034958)[4]. Genomic data for all B5/H24RxC isolates shown in this figure is in BioProject PRJNA951454 and Table S1.

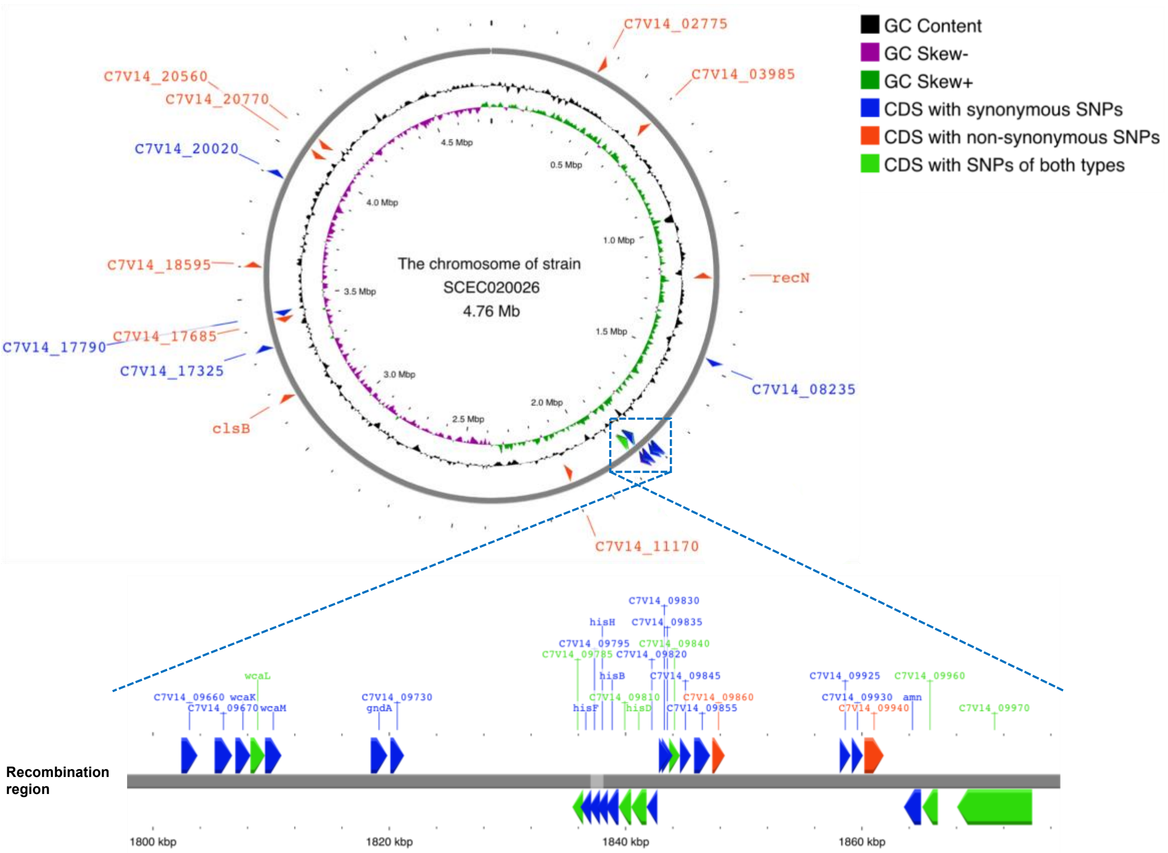

Fig. S7: Location of all synonymous and non-synomynous SNPs unique to the B5/H24RxC MDR clone in comparison to the B4/H24RxC MDR clone. The circular diagram was generated using Proksee (https://proksee.ca/). Detailed SNPs information is included in Supplementary Data 7.

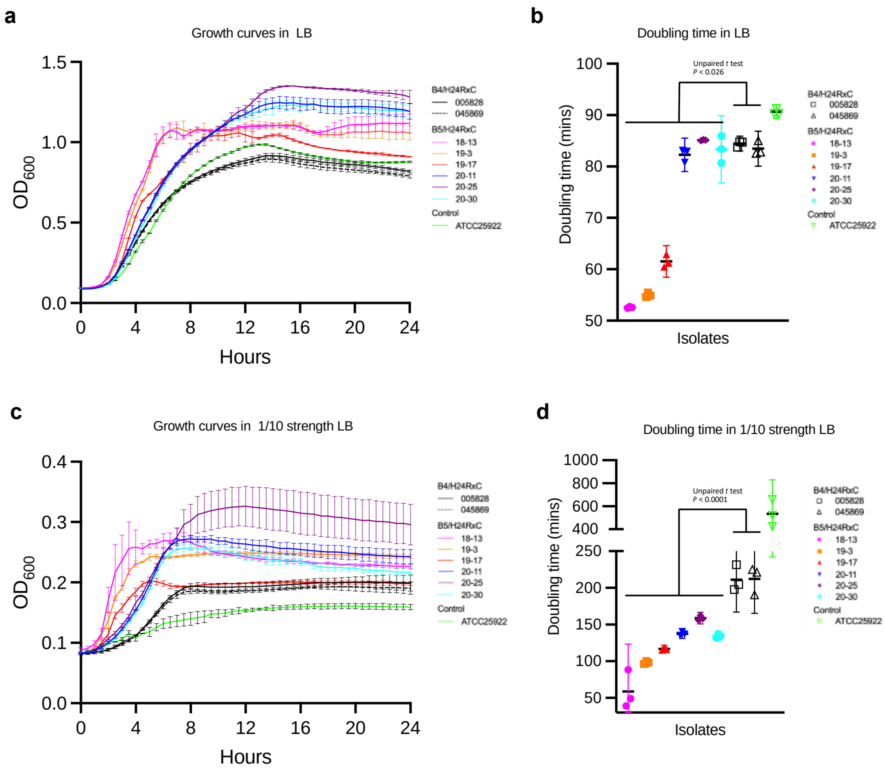

Fig. S8: Growth comparison of B4/H24RxC and B5/H24RxC clones. (a) Growth curves in full strength LB for strains of both clones. Data are shown as mean ± SD from n = 3 biological replicates. **(b)** Doubling time in full strength LB for strains of both clones. Statistical difference was assessed with two-tailed unpaired Student's *t* test. Data are shown as mean ± SD from n = 3 biological replicates. **(c)** Growth curves in 1/10 strength LB for strains of both clones. Data are shown as mean ± SD from n = 3 biological replicates. **(d)** Doubling time in 1/10 strength LB for strains of both clones. Statistical difference was assessed with two-tailed unpaired Student's *t* test. Data are shown as mean ± SD from n = 3 biological replicates. Strain ATCC 25922 was included as a growth control. Source data are provided as a Source Data file.

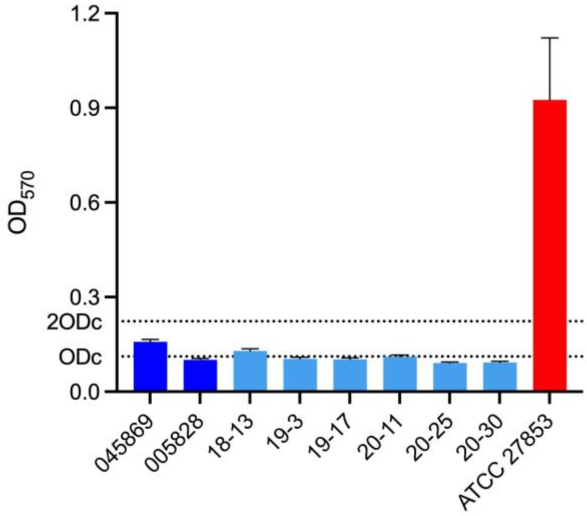

Fig. S9: Biofilm formation of isolates of both clones. A known biofilm former *Acinetobacter baumannii* strain ATCC 27853 was used as a positive control. The presented data are from a single representative experiment. Data are shown as mean ± SD from n = 12 technical replicates. ODc stands for optical density cutoff. Source data are provided as a Source Data file.

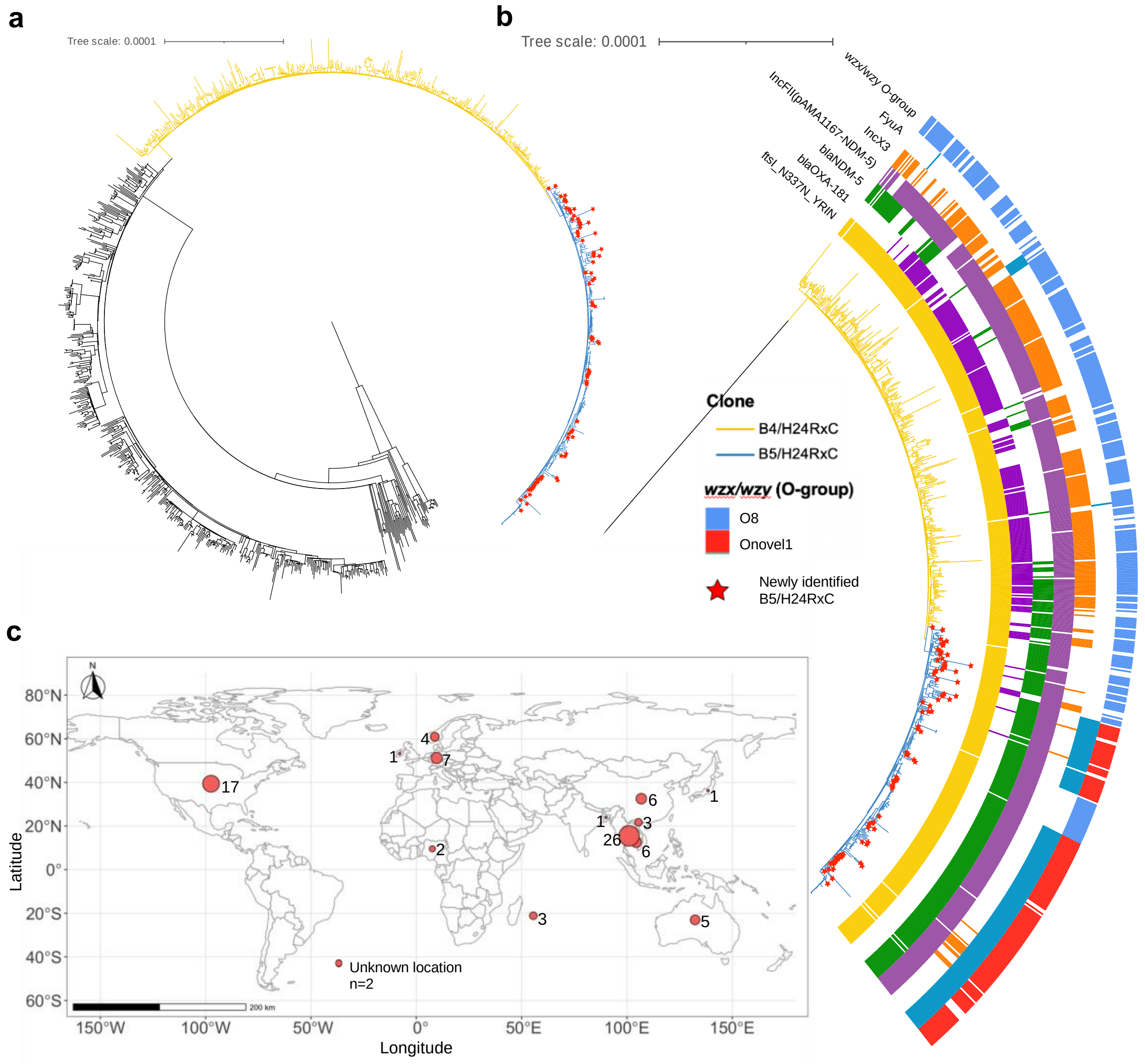

Fig. S10: Analysis on the recently available collection of ST410 (n=714) and B5/H24RxC clone (n=84). (a) Midpoint rooted maximum-likelihood phylogeny of 714 newly available ST410 genomes from EnteroBase (14 Jan 2022 to 27 Sept 2023) and 388 genomes (214 B4/H24RxC and 174 B5/H24RxC) from the original analysis, constructed using a core genome SNP alignment generated by Snippy v4.6.0 with strain 020026 (Genbank accessions CP034954 to CP034958) as the reference. Branch support was performed with 1,000 bootstrap replicates. **(b)** An enlarged phylogenetic tree showing B4/H24RxC and B5/H24/RxC clones with their genomic characteristics. **(c)** Global distribution of the newly identified B5/H24RxC isolates in the recent ST410 collection. Total number of B5/H24RxC isolates in each country is indicated next to the red circles. Source data are provided as a Source Data file.

Table S1: Genbank accessions for all B5/H24RxC isolates in BioProject PRJNA951454

Isolate	chromosome/plasmids	GenBank	Size (bp)	GC content (%)
E22	chromosome	CP123036.1	4,826,658	50.5
	pE22P1	CP123037.1	108,280	51.5
	pE22P2	CP123038.1	3,376	55
	pE22P3	CP123039.1	2,088	47
18-4	chromosome	CP123013.1	4,847,302	50.5
	p18-4P1	CP123014.1	107,858	51.5
	p18-4P2	CP123015.1	3,376	55
	p18-4P3	CP123016.1	2,088	47
20-16	chromosome	CP123024.1	4,844,449	50.5
	p20-16P1	CP123025.1	100,602	51.5
	p20-16P2	CP123026.1	3,373	55
	p20-16P3	CP123027.1	2,088	47
	p20-16P4	CP123028.1	1,552	51.5
19-7	chromosome	CP123017.1	4,838,085	50.5
	p19-7P1	CP123018.1	93,917	47.5
	p19-7P2	CP123019.1	83,900	50.5
	p19-7P3	CP123020.1	67,953	52.5
	p19-7P4	CP123021.1	3,376	55
	p19-7P5	CP123022.1	2,088	47
	p19-7P6	CP123023.1	1,552	51.5
20-20	chromosome	CP123029.1	4,799,026	50.5
	p20-20P1	CP123030.1	107,778	47
	p20-20P2	CP123031.1	84,132	50.5
	p20-20P3	CP123032.1	39,066	61.5
	p20-20P4	CP123033.1	3,376	55
	p20-20P5	CP123034.1	2,088	47
	p20-20P6	CP123035.1	1,552	51.5

Table S2: Doubling time (mins) for selected isolates of B4/H24RxC and B5/H24RxC in different strength of LB medium

	**B5/H24RxC**					
	**18-13**	**19-3**	**19-17**	**20-11**	**20-25**	**20-30**
**LB**	52.591± 0.146	55.032±0.433	61.527±1.241	82.284±1.307	85.124±0.109	83.348±2.627
**1/2 LB**	63.241±0.733	60.591±1.007	71.076±0.995	70.213±0.083	73.195±0.647	72.596±0.154
**1/10 LB**	58.763±26.031	98.218±2.264	116.800±1.918	137.436±2.601	158.813±3.176	134.506±2.511

	**B4/H24RxC**		**Control**
	**005828**	**045869**	**ATCC 25922**
**LB**	84.447±0.593	83.463±1.361	90.643±0.531
**1/2 LB**	82.715±1.251	84.354±2.012	86.875±2.074
**1/10 LB**	211.193±17.856	212.242±18.882	535.183±117.962

Table S3: Survival comparison analysis for wax moth larvae infected with different bacterial strains using Log-rank (Mantel-Cox) test.
B4/24RxC isolates (005828 and 045869); B5/24RxC isolates (18-13, 19-3, 19-17, 20-11, 20-25 and 20-30); Hypervirulent *Klebsiella pneumoniae* strain K1088; and hypervirulent *Acinetobacter baumannii* strain AB5075.

**Compared with 005828**	**Compared with 045869**	**Chi square**	**df**	**P value**	**P value summary**	**Are the survival curves sig different?**
18-13		11.06	1	0.0009	***	Yes
20-30		10.17	1	0.0014	**	Yes
20-25		11.58	1	0.0007	***	Yes
19-3		9.95	1	0.0016	**	Yes
20-11		11.28	1	0.0008	***	Yes
19-17		9.452	1	0.0021	**	Yes
	18-13	11.57	1	0.0007	***	Yes
	20-30	10.33	1	0.001	**	Yes
	20-25	11.12	1	<0.001	***	Yes
	19-3	9.853	1	0.002	**	Yes
	20-11	11.78	1	<0.001	***	Yes
	19-17	9.305	1	0.002	**	Yes
**Compared with K1088**	**Compared with AB5075**	**Chi square**	**df**	**P value**	**P value summary**	**Are the survival curves sig different?**
18-13		6.229	1	0.0126	*	Yes
20-30		5.284	1	0.0215	*	Yes
20-25		7.759	1	0.0053	**	Yes
19-3		5.297	1	0.0214	*	Yes
20-11		7.58	1	0.0059	**	Yes
19-17		4.935	1	0.0263	*	Yes
	18-13	1.494	1	0.2216	ns	No
	20-30	1.269	1	0.2599	ns	No
	20-25	2.372	1	0.1235	ns	No
	19-3	1.065	1	0.302	ns	No
	20-11	2.113	1	0.1461	ns	No
	19-17	0.645	1	0.4219	ns	No

Table S4: Nested sampling results summary for model selection.

Model	Population model	Molecular clock model	Marginal likelihood	SD	BF
GTR	skyline	Relaxed	-6708866.032	64.79	-
GTR	constant	Relaxed	-6708929.186	67.34	63.154
GTR	exponential	Relaxed	-6708985.186	66.42	119.154
GTR	skyline	Strict	-6709668.092	62.85	802.06
GTR	constant	Strict	-6709783.574	66.37	917.542
GTR	exponential	Strict	-6709826.161	63.91	960.129
HKY	skyline	Relaxed	-6709356.197	68.43	490.165
HKY	exponential	Relaxed	-6709358.375	63.27	492.343
HKY	constant	Relaxed	-6709579.835	66.33	713.803
HKY	skyline	Strict	-6710166.926	62.11	1300.894
HKY	exponential	Strict	-6710288.758	59.85	1422.726
HKY	constant	Strict	-6710299.953	64.835	1433.921

Supplementary References

1. Chen L, Peirano G, Kreiswirth BN, Devinney R, Pitout JDD. Acquisition of genomic elements were pivotal for the success of Escherichia coli ST410. *J Antimicrob Chemoth* 2022.
2. Roer L, Overballe-Petersen S, Hansen F, et al. Escherichia coil Sequence Type 410 Is Causing New International High-Risk Clones. *Msphere* 2018; **3**(4).
3. Hadfield J, Croucher NJ, Goater RJ, Abudahab K, Aanensen DM, Harris SR. Phandango: an interactive viewer for bacterial population genomics. *Bioinformatics* 2018; **34**(2): 292-3.
4. Feng Y, Liu L, Lin J, et al. Key evolutionary events in the emergence of a globally disseminated, carbapenem resistant clone in the Escherichia coli ST410 lineage. *Commun Biol* 2019; **2**: 322.
