# Supplementary material for: Global emergence of a hypervirulent carbapenem-resistant Escherichia coli ST410 clone
Source: Nat Commun. 2024 Jan 12;15:494. doi: 10.1038/s41467-023-43854-3 (PMC10786849; doi:10.1038/s41467-023-43854-3)
Supplement: Supplementary file 3 — Description of Additional Supplementary Files [file 41467_2023_43854_MOESM3_ESM.pdf]

<u>Description of Additional Supplementary Files</u>

Supplementary Data 1: Metadata for all Chinese CREC isolates collected in this study

Supplementary Data 2: Metadata for the global collection of ST410

Supplementary Data 3: Metadata for the *E.coli* ST410 isolated from the children's hospital in eastern China

Supplementary Data 4: AMR gene identification for the global collection of ST410 (n=956) using abritAMR pipeline

Supplementary Data 5: Pairwise core genome SNP distance (excluding recombination regions) for isolates of the B4/H24RxC and the B5/H24RxC MDR clones

Supplementary Data 6: Core-genome genes associated with the B5/H24RxC clone

Supplementary Data 7: Core-SNPs associated with the B5/H24RxC clone

Supplementary Data 8: Enterobase generated metadata table for *E. coli* ST410 made available from 14 Jan 2022 to 27 Sept 2023 (n=714)
