# Supplementary material for: Global emergence of a hypervirulent carbapenem-resistant Escherichia coli ST410 clone
Source: Nat Commun. 2024 Jan 12;15:494. doi: 10.1038/s41467-023-43854-3 (PMC10786849; doi:10.1038/s41467-023-43854-3)
Supplement: Supplementary file 12 — Reporting Summary [file 41467_2023_43854_MOESM12_ESM.pdf]

nature portfolio

Corresponding author(s): Mark Holmes

Last updated by author(s): 14 Nov 2023

Reporting Summary

Nature Portfolio wishes to improve the reproducibility of the work that we publish. This form provides structure for consistency and transparency in reporting. For further information on Nature Portfolio policies, see our Editorial Policies and the Editorial Policy Checklist.

Statistics

For all statistical analyses, confirm that the following items are present in the figure legend, table legend, main text, or Methods section.

n/a	Confirmed	
☐	☒	The exact sample size (*n*) for each experimental group/condition, given as a discrete number and unit of measurement
☐	☒	A statement on whether measurements were taken from distinct samples or whether the same sample was measured repeatedly
☐	☒	The statistical test(s) used AND whether they are one- or two-sided<br>*Only common tests should be described solely by name; describe more complex techniques in the Methods section.*
☒	☐	A description of all covariates tested
☒	☐	A description of any assumptions or corrections, such as tests of normality and adjustment for multiple comparisons
☐	☒	A full description of the statistical parameters including central tendency (e.g. means) or other basic estimates (e.g. regression coefficient) AND variation (e.g. standard deviation) or associated estimates of uncertainty (e.g. confidence intervals)
☐	☒	For null hypothesis testing, the test statistic (e.g. *F*, *t*, *r*) with confidence intervals, effect sizes, degrees of freedom and *P* value noted<br>*Give P values as exact values whenever suitable.*
☒	☐	For Bayesian analysis, information on the choice of priors and Markov chain Monte Carlo settings
☒	☐	For hierarchical and complex designs, identification of the appropriate level for tests and full reporting of outcomes
☒	☐	Estimates of effect sizes (e.g. Cohen's *d*, Pearson's *r*), indicating how they were calculated

Our web collection on statistics for biologists contains articles on many of the points above.

Software and code

Policy information about availability of computer code

Data collection	None used
Data analysis	Shovill v1.1.0; QUAST v5.0.2; CheckM v1.1.3; Centrifuge v1.0.4;<br>Unicycler v0.4.8; Prokka v1.14.0; ABRicate v1.0.1; FimTyper v1.0; abritAMR v1.0.13; MLST v2.19.0;<br>Snippy v4.6.0; Gubbins v2.4.1; SNP-sites v2.5.1; IQ-tree; iTOL; ggtree v3.2.1;<br>Fastbaps v1.0; snp-dists v0.8.2; Panaroo v1.2.7; Scoary v1.6.16;<br>Gene Construction Kit v4.5.1<br>web blastn<br>clinker v0.0.28<br>Treemmer v0.3<br>TempEst v1.5.3; Nested Sampling v1.1.0<br>BEAST v2.6.6; LogCombiner v2.6.6; Tracer v1.7.2; TreeAnnotator v2.6.6;<br>GraphPad Prism 9

For manuscripts utilizing custom algorithms or software that are central to the research but not yet described in published literature, software must be made available to editors and reviewers. We strongly encourage code deposition in a community repository (e.g. GitHub). See the Nature Portfolio guidelines for submitting code & software for further information.

Data

Policy information about availability of data

All manuscripts must include a data availability statement. This statement should provide the following information, where applicable:

- Accession codes, unique identifiers, or web links for publicly available datasets
- A description of any restrictions on data availability
- For clinical datasets or third party data, please ensure that the statement adheres to our policy

Sequence data and genome assemblies generated in this study have been submitted to GenBank under the BioProject PRJNA931432 and PRJNA951454. The individual Illumina sequence read accession numbers of ST410 isolates are listed in Supplementary Data 2. The accession numbers for the nanopore sequenced isolates in this study can be found in Table S1. Source data are provided with this paper.

Research involving human participants, their data, or biological material

Policy information about studies with human participants or human data. See also policy information about sex, gender (identity/presentation), and sexual orientation and race, ethnicity and racism.

Reporting on sex and gender	Sex and age (in age groups) was reported for 47 inpatients admitted to a children's hospital in eastern China between 2018 and 2020.
Reporting on race, ethnicity, or other socially relevant groupings	n/a
Population characteristics	Sex and age (in age groups) was reported for 47 inpatients admitted to a children's hospital in eastern China between 2018 and 2020.
Recruitment	n/a
Ethics oversight	This study was approved by the medical ethics committee of the First Affiliated Hospital of Guangzhou Medical University (GMU) on 21 May, 2018. For the work involving the CREC isolates from the children's hospital, ethics was approved by the medical ethics committee of the Children's Hospital of Soochow University on 5 January, 2021. Individual consent was obtained from the patients' guardians by hospital staff.

Note that full information on the approval of the study protocol must also be provided in the manuscript.

Field-specific reporting

Please select the one below that is the best fit for your research. If you are not sure, read the appropriate sections before making your selection.

☒ Life sciences ☐ Behavioural & social sciences ☐ Ecological, evolutionary & environmental sciences

For a reference copy of the document with all sections, see nature.com/documents/nr-reporting-summary-flat.pdf

Life sciences study design

All studies must disclose on these points even when the disclosure is negative.

Sample size	No statistical method was performed to predetermine sample size for our experiments. Instead, the sample sizes were chosen based on availability, practical considerations, previous similar studies in the field and specific purpose of each experiment.<br><br>A collection of 956 E. coli genomes were determined by the availability in our research labs and public database. For coalescent analysis of ST410 E. coli, Treemmer v0.3 was used to reduce the ST410 global phylogeny to 500 genomes, maintaining about 95% of the original genetic diversity.<br><br>Five isolates belong to the B5/H24RxC clone were selected based on their genomic differences, representing the range of diversity as suggested by their positions in the phylogeny.<br><br>For the genomic comparison of B4/H24RxC (n=214) and B5/H24RxC (n=174) clones, the number in each clone was determined by their presence in the total collection (n=956).<br><br>For Phenotypic assays, B4/24RxC isolates (n=2) and B5/24RxC isolates (n=6) were selected based on their availability in our research labs and their genetic characteristics.<br><br>For the wax moth infection model, each group contained 10 larvae. This is determined by our previous work and similar studies in the field.
Data exclusions	No data were excluded from analysis.

Replication	In general, experiments were replicated with two to three times in accordance to the generally accepted standard in the field. All attempted replications were successful similar results.
Randomization	Wax moth larvae were randomly allocated into different groups. Randomization was not relevant for other in vitro experiments as they did not involve allocation to different groups.
Blinding	For wax moth larvae infection assay, blinding was not required as the survival of wax larvae were not subjected to human bias. Blinding was not required for other in vitro experiments as they did not involve allocation to different groups.

Reporting for specific materials, systems and methods

We require information from authors about some types of materials, experimental systems and methods used in many studies. Here, indicate whether each material, system or method listed is relevant to your study. If you are not sure if a list item applies to your research, read the appropriate section before selecting a response.

Materials & experimental systems

n/a	Involved in the study
☒	☐ Antibodies
☒	☐ Eukaryotic cell lines
☒	☐ Palaeontology and archaeology
☐	☒ Animals and other organisms
☐	☒ Clinical data
☒	☐ Dual use research of concern
☒	☐ Plants

Methods

n/a	Involved in the study
☒	☐ ChIP-seq
☒	☐ Flow cytometry
☒	☐ MRI-based neuroimaging

Animals and other research organisms

Policy information about studies involving animals; ARRIVE guidelines recommended for reporting animal research, and Sex and Gender in Research

Laboratory animals	This study did not involve laboratory animals. This study used wax moth larvae. The larvae were about 35 days after hatching and of 250-350 mg (this information has been added in the Methods section).
Wild animals	This study did not involve wild animals.
Reporting on sex	Not relevant to wax moth larvae.
Field-collected samples	This study did not involve field-collected samples.
Ethics oversight	n/a

Note that full information on the approval of the study protocol must also be provided in the manuscript.

Clinical data

Policy information about clinical studies

All manuscripts should comply with the ICMJE guidelines for publication of clinical research and a completed CONSORT checklist must be included with all submissions.

Clinical trial registration	n/a
Study protocol	n/a
Data collection	Infection types and clinical complications information were collected from patients with carbapenem-resistant E. coli infection. Sex and age data (age groups) was collected for 47 inpatients admitted to a children's hospital in eastern China between 2018 and 2020.
Outcomes	n/a
